# *Lacticaseibacillus rhamnosus* LRJ-1 alleviates constipation through promoting gut *Bacteroides*-derived γ-aminobutyric acid production

**DOI:** 10.1016/j.crfs.2024.100924

**Published:** 2024-11-12

**Authors:** Tianqi Xia, Fuqing Huang, Fangfei Yun, Yayong Liu, Tianwei Wang, Siyue Wang, Sijie Jin, Xingwang Ma, Wenhan Wang, Jianzhuo He, Kunling Teng, Jin Zhong

**Affiliations:** a State Key Laboratory of Microbial Resources, Institute of Microbiology, Chinese Academy of Sciences, Beijing, China; b School of Life Science, University of Chinese Academy of Sciences, Beijing, China; c School of Life Sciences, Yunnan University, Kunming, China; d Chi Forest (Beijing) Food Technology Group Co., Ltd, Beijing, China

**Keywords:** Constipation, Gut microbiota, *Bacteroides*, γ-aminobutyric acid, *Lacticaseibacillus rhamnosus*

## Abstract

Multiple gastrointestinal disorders are associated with impaired gut microbiota. Probiotic *Lacticaseibacillus rhamnosus* can improve bowel disorder, however, the action mechanism is poorly understood. We integrated multi-omics data from the gut metagenome, metabolome, and colon transcriptome of constipated mice underlying *L. rhamnosus* LRJ-1 treatment to provide insights into host-microbial metabolic pathway. We found that oral administration of *L. rhamnosus* LRJ-1 alleviated constipation in mice accompanied by the increased abundances of fecal γ-aminobutyric acid (GABA) and intestinal commensal *Bacteroides*, and the activation of host GABAergic synapses. *B. uniformis* was the most enriched *Bacteroides* species in constipated mice treated with *L. rhamnosus* LRJ-1, and contributed to the increased abundance of GABA in the gut. Administration of either *B. uniformis* ATCC 8492 or GABA alleviated constipation and increased gastrointestinal motility in constipated mice. Knockout of the GABA biosynthetic *gad* gene in *B. uniformis* ATCC 8492 decreased GABA production and blocked its beneficial effects on constipation. These results confirm the therapeutic potential of *L. rhamnosus* LRJ-1 in alleviating constipation through promoting gut commensal *Bacteroides*-derived GABA production. Targeting the gut microbiome to regulate GABA production may open new insights for efficient constipation treatment.

## Highlights

• *Lacticaseibacillus rhamnosus* LRJ-1 alleviated constipation with the enrichment of *Bacteroides* and γ-aminobutyric acid (GABA).
• Transcriptomic analysis indicated the activated GABAergic synaptic pathway in colon was involved in constipation relief.
• GABA and *B. uniformis* instead of its GABA biosynthetic gene *gad* deficient strain are capable of alleviating constipation.
• A specific *L. rhamnosus* LRJ-1 - gut *Bacteroides* - host GABAergic synaptic pathway for constipation relief was discovered.

## Graphical abstract

**Figure:**
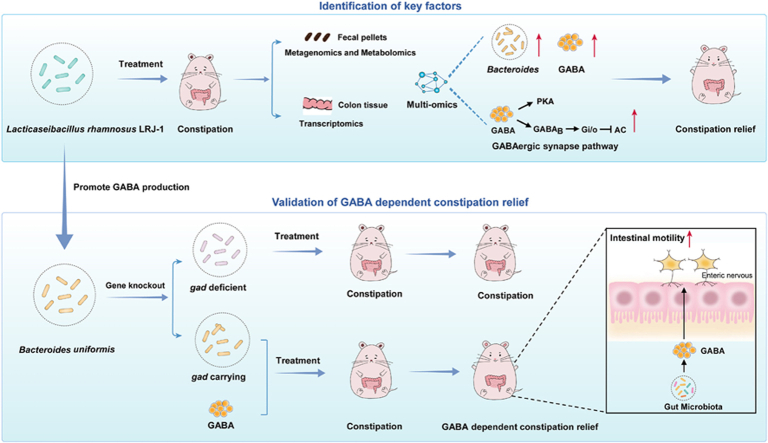

## 1 Introduction

Constipation is a globally prevalent gastrointestinal disorder affecting 14% of the global population (Barberio et al., 2021). It is characterized by dry feces, difficult evacuation, and is accompanied by recurrent discomfort, abdominal distension and restlessness (Bharucha and Lacy, 2020; Barberio et al., 2021). It influences the development of colorectal cancer (Power et al., 2013), Parkinson's disease (Savica et al., 2009) and mental disorder (Bresnahan et al., 2015). Preventing and managing constipation is crucial. Constipation pathogenesis is not yet fully understood and generally considered to result from multiple factors, including an overly refined diet or low dietary fiber intake, colon structural abnormalities, psychological factors such as depression and anxiety, gut microbiota imbalance, and medication effects (Rao and Go, 2010). Common treatments for constipation include dietary fiber, over-the-counter laxatives, intestinal secretagogues, and surgical interventions (Ramkumar and Rao, 2004). However, traditional treatments have some limitations. For example, dietary fiber is not effective for all patients with constipation. Long-term use of laxatives may lead to laxative-dependent colon affecting patient tolerance (Bharucha and Lacy, 2020). Therefore, there is an urgent need for safer and more effective treatments.

Previous study reported that colonization with gut microbiota increased colonic contractility but decreased gastrointestinal transit time in mice compared to germ-free controls (Kashyap et al., 2013). Impaired gastrointestinal motility has been implicated with a decrease in the level of beneficial microorganisms, e.g., *Bifidobacteria*, *Lactiplantibacillus*, *Lacticaseibacillus* and etc. (Khalif et al., 2005; Jomehzadeh et al., 2020). Therefore, orally administrated probiotics have been widely used to attenuate constipation for targeting the gut microbiome (Kim et al., 2021). To date, *Ligilactobacillus salivarius* (Qiu et al., 2022), *Lacticaseibacillus paracasei* (Cheng et al., 2023), *L*. *rhamnosus* (Wang et al., 2020a), *Lactiplantibacillus plantarum* (Ma et al., 2023) and *Pediococcus acidilactici* (Qiao et al., 2021), have been reported for improving loperamide-induced constipation in mice by modulating gut microbiota. Modifying the gut environments with probiotics may affect gastrointestinal motility and secretion and, hence, provide a protection for constipation. However, the action mechanism of how probiotic strain acts with gut microbes and the host to alleviate constipation are still relatively poorly understood.

Probiotic ingestion can lead to changes in microbial metabolites that interact with the host enteric nervous system (ENS), which regulates the gastrointestinal motility and secretion, and gut microbiota to ameliorate constipation (Dimidi et al., 2017). γ-aminobutyric acid (GABA) is a gut microbial-derived metabolite catalyzed by a glutamate decarboxylase (Gad) (Strandwitz et al., 2019). It is a major inhibitory neurotransmitter in the central nervous system (CNS), and regulates various physiological and psychological processes (Bravo et al., 2011). It calms nerve cells in the CNS and affects brain functions. Alterations in central GABA receptor expression are associated with the pathogenesis of depression which is usually comorbid with functional bowel disorders, such as constipation (Bravo et al., 2011). Furthermore, GABA locates throughout the gastrointestinal tract and is found in enteric nerves to influence gastrointestinal function (Hyland and Cryan, 2010). As a neuromodulator in ENS, GABA effecting in the gastrointestinal tract depends on the activation of ionotropic GABA_A_ receptors or metabotropic GABA_B_ receptors, resulting in a potential noteworthy regulation of the excitatory or inhibitory signaling in the ENS (Auteri, Zizzo and Serio 2015a; Auteri, Zizzo and Serio 2015b). Preclinical studies indicate the therapeutic effects of GABA or GABAergic drugs on autoimmune inflammation inhibitory (Bhat et al., 2010), antimicrobial host defenses (Kim et al., 2018) and emotional behavior regulation (Bravo et al., 2011). However, it is poorly understood whether and how GABA or GABAergic signaling regulates constipation.

This study aimed to identify and systematically validate the key microbial-derived products and the corresponding gut microbiota and host alteration affected by a human gut-originated *L*. *rhamnosus* LRJ-1 for alleviating constipation. First, we performed multi-omics analysis including fecal metabolomic, microbial metagenomic and host transcriptomic to investigate the alteration of metabolites and host enzymes driven by altered microbial metabolism, which corresponded with concurrent changes in host physiology. Then, we identified a *Bacteroides*-derived GABA responsible for the effective alleviation of constipation in the mice model and further validated its functional roles by either a gene-knockout *Bacteroides* strain or GABA treatment. In conclusion, we evaluated the therapeutic effects of *L*. *rhamnosus* LRJ-1 on loperamide-induced constipation, and identified a beneficial gut *Bacteroides*-derived GABA interacting with host GABAergic synapses to affect gastrointestinal motility. Our study confirmed the therapeutic potential of the gut microbial-derived GABA for gastrointestinal diseases, and identified how an exogenous probiotic interacts with a gut commensal microbiota to regulate host physiology.

## 2 Materials and methods

### 2.1 Bacteria strains and culture condition

*L*. *rhamnosus* LRJ-1 was isolated from healthy human gut and incubated anaerobically in De Man-Rogosa - Sharpe (MRS) medium at 37 °C. MRS (CM1175, Oxoid, UK) consists of peptone (10.0 g/L), “Lab-Lemco" powder (8.0 g/L), yeast extract (4.0 g/L), glucose (20.0 g/L), sorbitan mono-oleate (1 mL), di-potassium hydrogen phosphate (2.0 g/L), sodium acetate 3H_2_O (5.0 g/L), Tri-ammonium citrate (2.0 g/L), magnesium sulphate·7H_2_O (0.2 g/L), and manganese sulphate·4H_2_O (0.05 g/L).

*Bacteroides uniformis* ATCC 8492 which was isolated from healthy human stool was obtained from professor Lei Dai in Shenzhen Institute of Advanced Technology, Chinese Academy of Sciences. The CRISPR-Cas genome-editing tool in *B*. *uniformis* ATCC 8492 (BU) was constructed using a previously established method (Zheng et al., 2022) and BUΔ*gad* was constructed by CRISPR-Cas System. BU and its derivatives - BUΔ*gad* were incubated anaerobically at 37 °C in BHIchv. BHI (Brian Heart Infusion, CM1135B, Oxoid, UK) consists of brain infusion solids (12.5 g/L), beef heart infusion solids (5.0 g/L), protease peptone (10.0 g/L), glucose (2.0 g/L), sodium chloride (5.0 g/L) and disodium phosphate (2.5 g/L). BHIchv consists of BHI medium supplemented with cysteine (0.5 g/L), vitamin K_3_ (0.5 mg/L), and hemin (5 mg/L).

### 2.2 Animal experiment: The role of *L. rhamnosus* LRJ-1 on constipation relief

Female SPF Balb/c mice (8 weeks old) were purchased from Beijing Vital River Laboratory Animal Technology Co., Ltd. The mice were housed under standard environmental conditions of 22 °C ± 2 °C and 50% ± 5% relative humidity with a 12 h light-dark cycle. The feed used in this study was purchased from SPF (Beijing) Biotechnology Co., Ltd., specifically the maintenance feed for mice (SPF-F02-002). The feed formula includes corn, soybean meal, fish meal, dicalcium phosphate, various vitamins, trace elements, and amino acids. The fiber content of this feed is 20 g/kg. To evaluate the effects of *L. rhamnosus* LRJ-1 on loperamide-induced constipation, mice were randomly divided into three groups (n = 11): the Con group, Lop group and LRJ-1 group. The mice were administered a gavage dose of loperamide (10 mg kg^−1^) to induce constipation, mice were treated with *L. rhamnosus* LRJ-1 at a sufficient dose (5 × 10^8^ CFU per day) as LRJ-1 group, or sterilized PBS (0.01 M, pH 7.4) as Lop group. Moreover, the mice were given only sterilized PBS without loperamide administration as Con group. At the conclusion of the animal experiment, the mice were humanely anesthetized by isoflurane. Subsequently, the eyeballs were removed and the blood was collected quickly. Following these procedures, the mice were euthanized promptly to prevent it from experiencing unnecessary suffering.

### 2.3 Measurement of fecal pellet water content

The number of fecal pellets for per cage was recorded in a 5 h experiment for gut motility measurement of mice. The fresh fecal pellets from per cage were collected in a separate sterile EP tube. After obtaining the wet weight, each sample was subject to a freeze dryer for 48 h to obtain the dry weight.

### 2.4 Enzyme-linked immunosorbent assay

The contents of motilin (MTL) (LV30625), somatostatin (SS) (LV30626), substance P (SP) (LV30596), and vasoactive intestinal peptide (VIP) (LV30597) in serum were determined using ELISA Kits according to instructions.

### 2.5 Fecal metabolic profiling analysis

To extract the metabolites, an appropriate amount of sample was added to the precooled methanol/acetonitrile/water solution (2:2:1, v/v), and sonicated for 30 min. The mixture was centrifuged and the supernatant was dried. Then the dried samples were re-dissolved in 100 μL acetonitrile/water (1:1, v/v) solvent for analysis. The LC-MS analysis was performed by Shanghai Applied Protein Technology Co., Ltd. Briefly, each sample was injected onto the UHPLC (Agilent 1290 Infinity LC) coupled to a quadrupole time of flight (AB Sciex TripleTOF 6600) for MS acquisition. The raw MS data were converted to MzXML files by ProteoWizard MSConvert and then imported into XCMS software for peak picking. Subsequently, compound identification was performed with an in house database (Shanghai Applied Protein Technology) established with available authentic standards. Finally, the processed data were analyzed by Student's *t*-test to determine the metabolites significance (VIP >1 and p value < 0.05) of differences between two groups. Metabolic pathway analysis was performed in MetaboAnalyst 5.0.

### 2.6 Fecal metagenomic sequencing and data processing

The DNA was extracted using the E.Z.N.A.® Soil DNA Kit (Omega Bio-tek, USA). The metagenomic analysis was performed by Shanghai Applied Protein Technology Co., Ltd. Briefly, a total amount of 1 μg DNA per sample was prepared for library construction. Sequencing libraries were constructed using NEBNext® Ultra™ DNA Library Prep Kit for Illumina (NEB, USA) following manufacturer's recommendations. These were sequenced on an Illumina NovaSeq platform, and paired-end reads were generated. The Raw Data acquired was processed by Readfq to obtain the Clean Data. Then, the Clean Data is assembled and analyzed by SOAPdenovo software. The Clean Data of each sample is matched to initial gene catalogue by Bowtie2.2.4, and get the number of reads to which genes mapped in each sample. The Unigenes is blast to the sequences of NR database of NCBI using DIAMOND software, followed by taxonomic identification with MEGAN. Adopt DIAMOND software is used to blast Unigenes to KEGG database, and the best Blast Hit is used for subsequent analysis.

### 2.7 Colon transcriptomic sequencing and data processing

The total RNA of colon was extracted using RNAsimple Total RNA Extraction Kit (TIANGEN) according the manufacturer's instructions. DNase I (TaKaRa) was used to remove genomic DNA. The RNA quality was assessed using 2100 Bioanalyser (Agilent) and quantified using the ND-2000 (NanoDrop Technologies). The RNA-seq transcriptome analysis was performed by Shanghai Applied Protein Technology Co., Ltd. Briefly, 1 μg of total RNA was used for preparation of the RNA-seq transcriptome library. After quantified by TBS380, the paired-end RNA-seq sequencing library was sequenced with the Illumina HiSeq xten/NovaSeq 6000 sequencer. The obtained raw data underwent quality controlled to obtain clean reads, which were aligned separately to the reference genome using HISAT2 software. The mapped reads of each sample were assembled by StringTie software. Then, RSEM software was used to quantify gene abundances and KOBAS software was used to perform KEGG pathway analysis.

### 2.8 Animal experiment: The role of *B. uniformis* and GABA on constipation relief

Female SPF Balb/c mice (8 weeks old) were purchased from Beijing Hfk Bioscience Co., Ltd. The animal experiment was performed as previously described (Zhang et al., 2023). Briefly, the BU was dissolved in PBS and administered to the mice (n = 10, 5 × 10^8^ CFU per day) via oral gavage using a gavage needle as well as BUΔ*gad* (5 × 10^8^ CFU per day). GABA was also dissolved in PBS. The mice (n = 10) were intraperitoneal injected with GABA (LGA: 2 mM, 100 μL per mouse; HGA: 20 mM, 100 μL per mouse) using a syringe. After 14 days, the effects of *B*. *uniformis* and GABA on loperamide-induced constipation in mice were assessed.

### 2.9 Determination of small intestinal transit

At the endpoint of animal experiment, mice fasted overnight while water was provided. All mice were given oral gavage of 200 μL of 0.5% activated charcoal solution in combination with sodium carboxymethyl cellulose powder. After 20 min, the abdomen of each mouse was opened, and the entire small intestine (from the pylorus to the cecum) was carefully taken out and placed it on tray. The distance moved by the activated charcoal and the total length of the small intestine were measured. The small intestinal transit of each mouse was calculated as the percentage of the moving distance of the activated charcoal powder relative to the total length of the small intestine.

### 2.10 Tissue section staining

Fresh colonic tissue was fixed in 4% paraformaldehyde for more than 24 h. The tissues were embedded in paraffin wax using an embedding machine (JB-P5, Wuhan Junjie Electronics Co., Ltd.). Subsequently, tissue sections of approximately 5 μm thickness were obtained using a pathological slicer (RM2016, Shanghai Leica Instrument Co.). Hematoxylin and eosin (HE) or Alcian Blue staining was then performed on the tissue sections. The stained tissues were observed under an optical microscope (Nikon Eclipse E100) using an imaging system (Nikon DS-U3).

### 2.11 Transcriptional activity of glutamate decarboxylase in *B. uniformis* ATCC 8492 effected by *L*. *rhamnosus* LRJ-1

Before co-culture, BU was grown in BHIchv broth medium and *L. rhamnosus* LRJ-1 was grown in a MRS broth medium and washed in PBS before being used to inoculate BHIchv. Bacteria were harvested at the mid log phase. The cells were collected via centrifugation (6000 g, 3 min) and ground with liquid nitrogen. Total RNA was extracted using TaKaRa MiniBEST Universal RNA Extraction Kit (9767) according to the instruction. The cDNA was synthesized using cDNA Synthesis Kit (TaKaRa, RR036A). qRT-PCR was performed in a 96-well plate following the Bestar® SybrGreen qPCR mastermix (DBI-2043) instruction. The specific primers were designed using NCBI Primer-BLAST. The 16S rDNA was used as a reference gene.

Primers for Gad of BU (F: 5′-GTGGGTACTCTCCATCAGCG-3′, R: 5′-TGGCACCGAGGTAGTTTACG-3′). Primers for 16S of BU (F: 5′-GGCTTACCATGCAGTCGAGG-3′, R: 5′-GAGTCATCGGCAGGTTGGAT-3′).

### 2.12 Quantification of GABA production

Different concentrations (0.15 mM, 0.30 mM, 0.61 mM, 1.21 mM, 2.42 mM, 4.85 mM) of GABA (Sigma-Aldrich) standards were prepared and filtered through a 0.2 μm filter. Then these standards were transferred directly into LC-MS vials. An aliquot of each standard (5 μL) was injected onto the LC-MS (Agilent 1260) and separated by 5 μm C18-AQ 4.6 × 250 mm column with flow rate of 1 mL/min. MS were acquired with an Agilent Accurate-Mass-Q-TOF MS 6520 system equipped with an Electrospray ionization (ESI) source using the negative ionization mode. A standard curve with GABA concentration as the abscissa and peak area as the ordinate was obtained: y = 53712x+601.53 (R^2^ = 0.9954). To measure the impact of Gad on the GABA production of BU, triplicate cultures of BU and BUΔ*gad* were grown in 3 mL BHIchv anaerobically for 48 h, the cells were centrifuged, and the supernatant was filtered through a 0.2 μm filter. Then these samples were separated following the same program as used above. The GABA standard curve was used as a reference to calculate the yield of GABA of BU and BUΔ*gad*.

### 2.13 Detection of fecal short chain fatty acids

The fecal samples were ground and centrifuged (18000 g, 20 min, 4 °C). The supernatant was sonicated with sulphuric acid (50%) and diethyl ether for short chain fatty acids (SCFAs) extraction. The diethyl ether layer of the supernatant was passed through anhydrous sodium sulphate for determination. The GC-MS analysis was performed using a gas chromatography-time-of-flight mass spectrometry system (GC-TOFMS, Leco Corp.) by Shanghai Majorbio Bio-Pharm Technology Co., Ltd. The raw data were analyzed by ChromaTOF software (v5.51, Leco corp., USA) for peak integration, correction and quantification of each metabolite.

### 2.14 Statistical analyses

The data were represented using mean ± standard error. The statistical analyses were performed with GraphPad Prism 8 and the OmicStudio tools at https://www.omicstudio.cn/tool.

## 3 Results

### 3.1 *L. rhamnosus* LRJ-1 protect mice from constipation

**Fig. 1:**
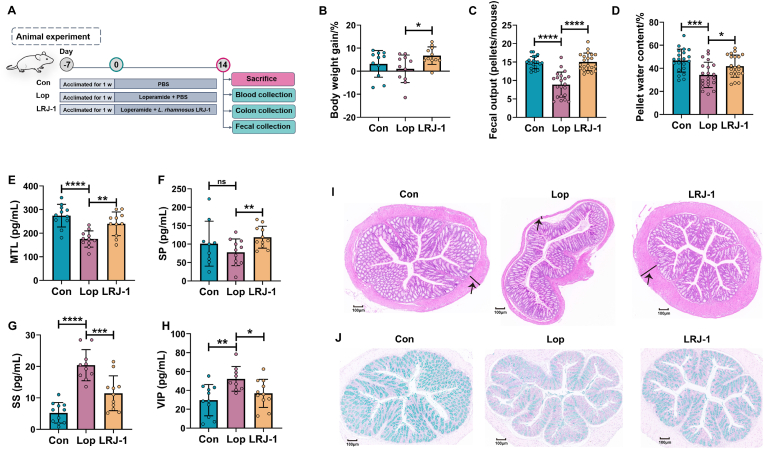
***L. rhamnosus* LRJ-1 alleviates loperamide-induced constipation in mice.** (A) The experimental design. The picture of the mouse was created with BioRender.com. (B) The changes in body weight for each mouse during 2 weeks treatment. (C) Average number of feces eliminated per mouse. (D) The effect of *L. rhamnosus* LRJ-1 on pellet water content. (E) The effect of *L. rhamnosus* LRJ-1 on serum motilin (MTL). (F) The effect of *L. rhamnosus* LRJ-1on substance P (SP). (G) The effect of *L. rhamnosus* LRJ-1 on vasoactive intestinal peptide (VIP). (H) The effect of *L. rhamnosus* LRJ-1on serum somatostatin (SS). (I) The effect of *L. rhamnosus* LRJ-1 on histological morphology of colon by hematoxylin & eosin staining. (J) The effect of *L. rhamnosus* LRJ-1 on colonic mucin by alcian blue staining. Con: control group; LOP: loperamide treated mice group; LRJ-1: *L. rhamnosus* LRJ-1 treated constipation mice group. ∗p < 0.05, ∗∗p < 0.01, ∗∗∗p < 0.001, ∗∗∗∗p < 0.0001 as determined by one-way ANOVA (B–D) or Wilcoxon signed-rank test (E–H). The black arrows indicate the muscle thickness.

To investigate the effect of *L. rhamnosus* LRJ-1 on constipation, loperamide-induced constipated mice were administered with *L. rhamnosus* LRJ-1 for two weeks as depicted in Fig. 1A. Results showed that *L. rhamnosus* LRJ-1 treatment significantly increased the body weight gain of constipation mice, with an average of 5.6-fold increase compared to the model group (Fig. 1B). Both fecal pellets number and pellet water content in the loperamide-induced constipation mice were lower in comparison to the control group. However, *L. rhamnosus* LRJ-1 treatment remarkably increased the fecal pellets number and pellet water content of the constipation mice (Fig. 1C, D). These findings indicated that *L. rhamnosus* LRJ-1 has a beneficial effect on alleviating constipation. Moreover, we tested the serum levels of neurotransmitters which are associated with gastrointestinal peristalsis. We found that *L. rhamnosus* LRJ-1 effectively up-regulated the levels of excitatory neurotransmitters substance P (SP) and motilin (MTL) (Fig. 1E, F), and down-regulated the levels of inhibitory neurotransmitters somatostatin (SS) and vasoactive intestinal peptide (VIP) in constipated mice (Fig. 1G, H), indicating that *L. rhamnosus* LRJ-1 may promote the gastrointestinal peristalsis.

To further examine the mitigatory effect of *L. rhamnosus* LRJ-1 on constipation in mice, histological staining was employed to assess the changes of intestinal morphology, mucin and gut barrier. The colonic histomorphology in Con group showed an intact intestinal structure, while that of a thinner muscle layer was observed in constipated mice from the Lop group (as indicated by black arrows in Fig. 1I). The muscle layer thickness and colonic morphology of constipated mice were recovered to normal status under *L. rhamnosus* LRJ-1 administration (Fig. 1I). Furthermore, the number of goblet cells decreased in the Lop group compared to the control group, but significantly increased after the mice were treated with *L. rhamnosus* LRJ-1 (Fig. 1J). Analysis of intestinal tissue further revealed the protective effect of *L. rhamnosus* LRJ-1 on constipation mice.

### 3.2 *L. rhamnosus* LRJ-1 shapes metabolites composition accompanied by increased level of fecal GABA

**Fig. 2:**
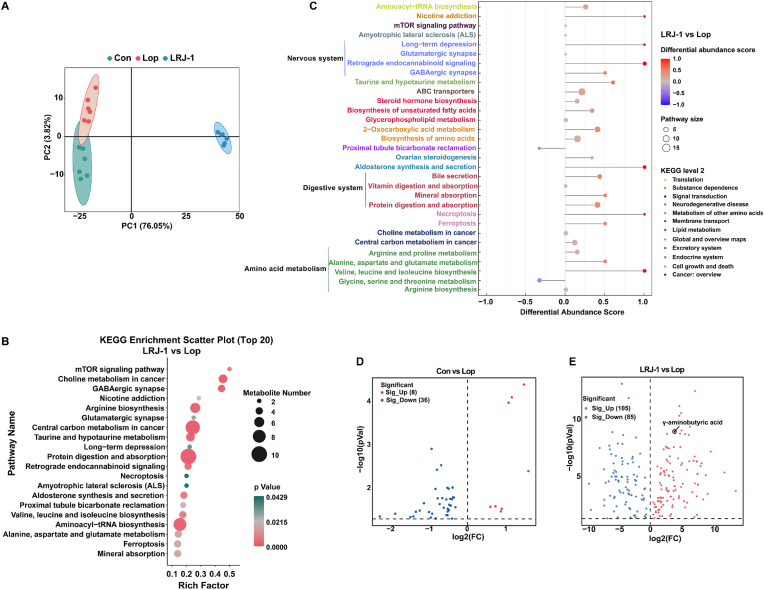
***L. rhamnosus* LRJ-1 shapes metabolites composition accompanied by increased level of fecal GABA in constipated mice**. (A) PLS-DA score plot of metabolites in Con, Lop and LRJ-1 groups. (B) KEGG pathway enrichment analysis of differential metabolites (LRJ-1 vs Lop group). (C) A pathway-based analysis of metabolic changes (LRJ-1 vs Lop group). The differential abundance score captures the gross changes for all identified metabolites in the pathway. A score of 1 indicates all identified metabolites in the pathway are upregulated, while that of −1 indicates downregulated. (D) Volcano plot of metabolites of Con vs Lop group. (E) Volcano plot of metabolites of LRJ-1 vs Lop group. p value < 0.05. Bioinformatic analysis was performed with the OmicStudio tool at https://www.omicstudio.cn/tool.

Metabolites of gut microbiota have been demonstrated that they can affect intestinal motility (Dimidi et al., 2017). To identify metabolic changes responsive to *L. rhamnosus* LRJ-1 administration, a metabolic profiling of fecal samples was performed. The partial least squares discriminant analysis (PLS-DA) revealed that the metabolites composition in both *L. rhamnosus* LRJ-1 administered mice and control mice were significantly different from that of constipated mice (Fig. 2A). Furthermore, metabolites regulated by *L. rhamnosus* LRJ-1 are mainly enriched in KEGG pathways including mTOR signaling pathway, choline metabolism in cancer, GABAergic synapse, etc. (Fig. 2B). To further determine the alteration of metabolic pathways, differential abundance score plot of pathways was analyzed. Most of the pathways were upregulated under *L. rhamnosus* LRJ-1 treatment, except for Proximal tubule bicarbonate reclamation and Glycine, serine and threonine metabolism (Fig. 2C). The nervous system, digestive system and amino acid metabolism had the highest number of pathway upregulations (Fig. 2C). Among them, the enriched GABAergic synapse (Top 3) was also upregulated. The alteration of differential metabolites is represented as a volcano plot. Indeed, we found that GABA was also significantly upregulated by *L. rhamnosus* LRJ-1, which belongs to the GABAergic synapse. Thus, we assume that the increase of GABA by *L. rhamnosus* LRJ-1 plays important roles in the therapeutic effects on constipation.

### 3.3 *L. rhamnosus* LRJ-1 protects mice from constipation by elevating *Bacteroides* level in the gut microbiota

**Fig. 3:**
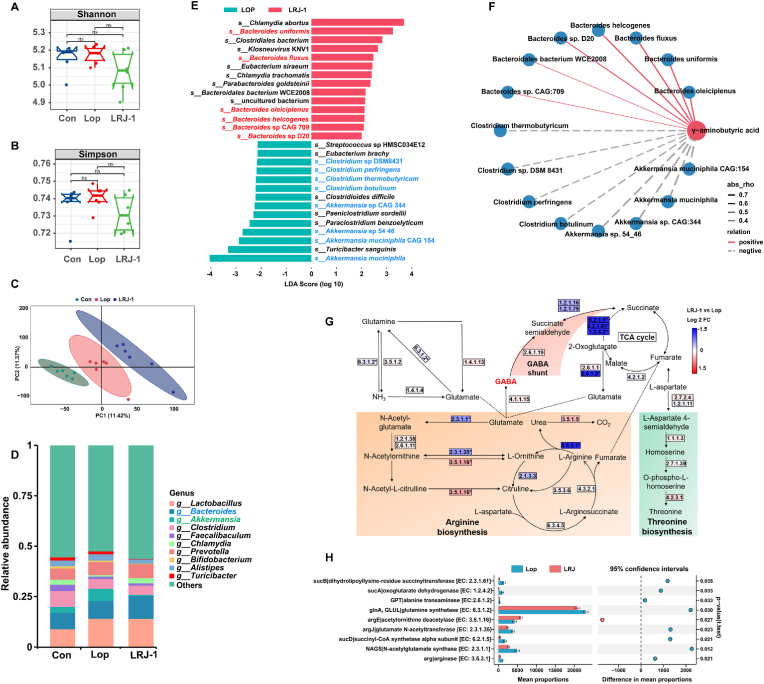
***L. rhamnosus* LRJ-1 elevated *Bacteroides* level in the gut microbiota and the level of various enzymes.** (A and B) Boxplots of alpha diversity indices of bacterial community, include Shannon (A) and Simpson (B) indices. (C) PLS-DA score plot of bacterial community in Con, Lop and LRJ-1 groups. (D) Change in genus with different treatments. (E) The log-transformed LDA scores computed with LEfSe for bacterial taxa differentially abundant between LRJ-1 and Lop groups. Positive and negative LDA score indicates enrichment in LRJ-1 and Lop group, respectively. (F) Correlation between GABA and microbes was estimated by Spearman's correlation analysis. (G) Representation of GABA synthesis related metabolic pathways identified from metagenomics data. Pathways include GABA shunt, arginine biosynthesis, threonine biosynthesis and TCA cycle. (H) The abundance difference of enzyme genes involved in GABA shunt, arginine biosynthesis, threonine biosynthesis and TCA cycle pathway between LRJ-1 and Lop groups was analyzed by STAMP. Bioinformatic analysis was performed with the OmicStudio tool at https://www.omicstudio.cn/tool and software STAMP v2.1.3.

Metabolic phenotyping reflected the gut microbiota activity on alleviation of constipation. To further explore the effect of *L. rhamnosus* LRJ-1 on gut microbiota, the metagenomic analysis of feces was performed. The Shannon and Simpson index showed no significant differences in OTU diversity between groups (Fig. 3A, B). However, PLS-DA analysis revealed distinct structure of the gut microbiota between LRJ-1 group and Con group in the first component (Fig. 3C). Taxon-based analysis revealed changes in the gut microbial composition. At the genus level, *L. rhamnosus* LRJ-1-treatment obviously enhanced the abundance of *Bacteroides* but reduced *Akkermansia* abundance in constipated mice (Fig. 3D). The analysis of linear discriminant analysis effect size (LEfSe) revealed marked changes at the species level. *L. rhamnosus* LRJ-1-treatment significantly enriched 14 species and depleted 14 species (Fig. 3E). It is worth noting that about half of the enriched species belong to *Bacteroides* genera, including *Bacteroides uniformis, B*. *fluxus, B*. *oleiciplenus* and so on. Most of depleted species belongs to *Akkermansia* and *Clostridum* genera (Fig. 3E). Correlation analysis revealed that the abundances of *Bacteroides* was positively correlated with the concentrations of GABA, whereas *Akkermansia* and *Clostridum* were negatively correlated with it (Fig. 3F). The enriched *Bacteroides* spp., including *B*. *fluxus*, *B. uniformis*, *B. helcgenes*, and *B. oleiciplenus* were predicted to be the main contributors to the increased level of gut GABA.

A previous study reported that *Bacteroides* ssp. produce large quantities of GABA and are perhaps the major GABA-producing bacteria in human gut (Strandwitz et al., 2019). We suggested that the increase in GABA caused by *L. rhamnosus* LRJ-1 might be related to the enrichment of *Bacteroides* levels. Hence, the Kyoto Encyclopedia of Genes and Genomes (KEGG) pathways of microbial genes were analyzed to gain genetic insights of the increased GABA production. GABA metabolism involves the participation of several metabolic pathways including the GABA shunt, TCA cycle, glutamine biosynthesis, arginine biosynthesis, and threonine biosynthesis (Fig. 3G). We found a significant decrease in catalase [EC 6.3.1.2] in feces of mice with *L. rhamnosus* LRJ-1 treatment, which is responsible for the production of glutamine from glutamate, and no significant difference in the reverse enzyme-catalyzed catalyze glutamine to glutamate [EC 1.4.1.13, EC 3.5.1.2 and EC 1.4.1.14] was observed (Fig. 3G, H). Furthermore, we also found a significant decrease in catalase [EC 2.3.1.1], which consumes glutamate to produce N-Acetyl-glutamate. This may promote the accumulation of glutamate, a GABA precursor, which is then catalyzed by Gad and leads to elevated level of GABA. Combining these results, we deduced that the gut symbiont *Bacteroides* were enriched by *L. rhamnosus* LRJ-1 and promoted the GABA production to relief constipation.

### 3.4 *L. rhamnosus* LRJ-1 enhanced GABAergic synapses in constipated mice

**Fig. 4:**
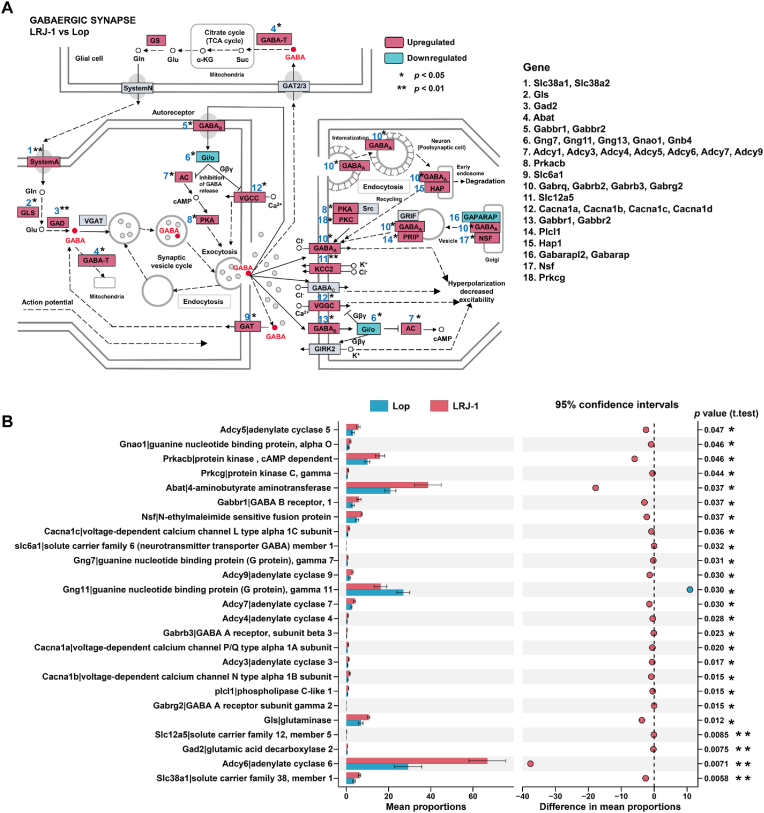
***L. rhamnosus* LRJ-1 enhanced GABAergic synapses in colon of constipated mice.** (A) KEGG map of regulation of the GABAergic synapse pathway, with each block representing a group of genes. Blue/red backgrounds indicate significantly decreased/increased abundance of genes, respectively. Differentially expressed genes in each block are highlighted on the right of the boxes. (B) The differences of genes related to GABAergic synapse pathway between LRJ-1 and Lop groups was analyzed by STAMP. The data was performed by software STAMP v2.1.3.

Multiple diseases are associated with an altered GABAergic profile, which has been reported to modulate gastrointestinal motility and secretory (Luscher et al., 2011; Auteri et al., 2015a). Pathways involving in GABAergic synapse was highlighted in metabolomics results (Fig. 2B). To further investigate the link between the host GABAergic pathway and constipation underlying the effect of *L. rhamnosus* LRJ-1, a colonic transcriptome analysis was done. GABA is packaged into vesicles and released into the synaptic cleft where it binds to target receptors on the postsynaptic surface (Fig. 4A). The result of colonic transcriptome analysis showed that except for Gng11 (involved in Gi/o), most of the expression levels of genes involved in GABAergic pathway were significantly upregulated in LRJ-1 treated mice compared to untreated constipated mice (Fig. 4A, B). Among these changes, the decreased Gi/o expression level and the increased expression levels of GABA_B_, AC and PKA in LRJ-1 group may be responsible for promoting GABA release in the host colon (Fig. 4A). These results indicate that the elevated abundance of GABA caused by *L. rhamnosus* LRJ-1 treatment activate GABAergic synapse pathway in host to attenuate constipation.

### 3.5 GABA ameliorates constipation in mice

**Fig. 5:**
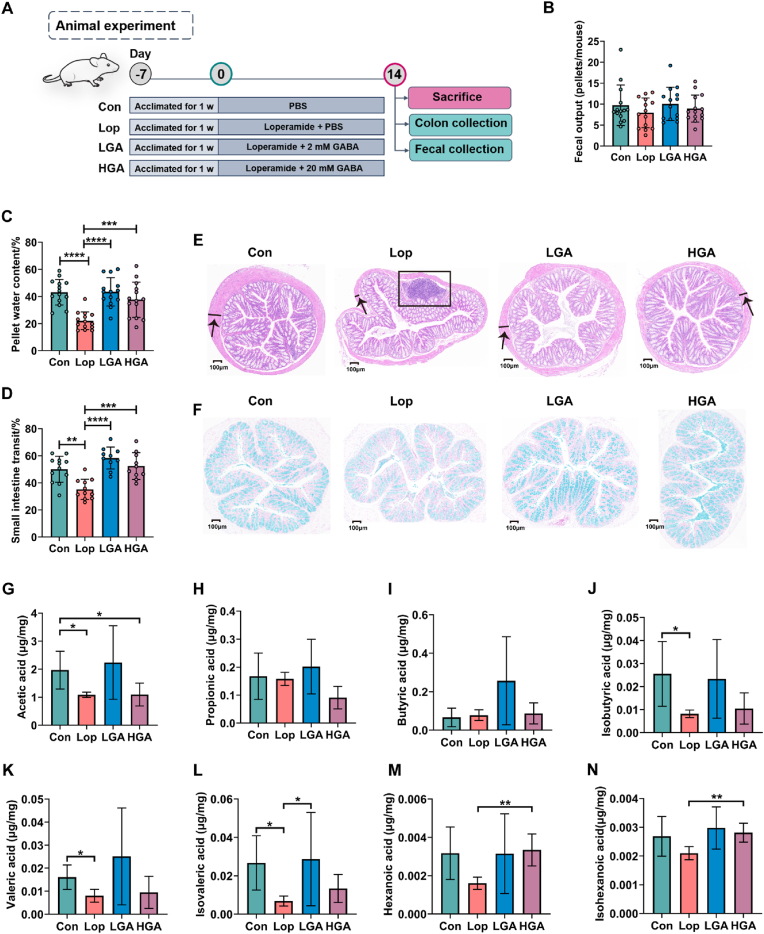
**GABA enriched by *L. rhamnosus* LRJ-1 can ameliorate constipation in mice**. (A) The experimental design. The picture of the mouse was created with BioRender.com. (B–D) The effect of lower concentration of GABA (LGA) with 2 mM and higher concentration of GABA (HGA) with 20 mM on (B) fecal output, (C) pellet water content, (D) small intestine transit in loperamide induced constipated mice. (E) The effect of LGA and HGA on histological morphology of colon by hematoxylin & eosin staining. (F) The effect of LGA and HGA on colonic mucin by alcian blue staining. (G–N) The effect of LGA and HGA on the contents of fecal short chain fatty acids by GC/MS, including (G) acetic acid, (H) propionic acid, (I) butyric acid, (J) isobutyric acid, (K) valeric acid, (L) isocaleric acid, (M) hexanoic acid and (N) isohexanoic acid. Con: control; LOP: loperamide; LGA: lower concentration of GABA. HGA: higher concentration of GABA. ∗p < 0.05, ∗∗p < 0.01, ∗∗∗p < 0.001, ∗∗∗∗p < 0.0001 as determined by one-way ANOVA (B–D) or Wilcoxon signed-rank test (G–N). The black arrows indicate the muscle thickness. The black box indicates the inflammatory infiltration.

Next, we sought to profile the GABA-modulating potential in constipation. The lower concentration GABA (LGA, 2 mM) and higher concentration GABA (HGA, 20 mM) were separately administered to constipated mice (Fig. 5A). Both LGA and HGA significantly increased pellet water content (Fig. 5C), reversed small intestine transit (Fig. 5D), reduced colonic pathology (Fig. 5E), and increased goblet cells (Fig. 5F) in constipated mice, suggesting that GABA are capable of alleviating constipation. We also found that GABA administration led to the alteration of SCFAs which have been reported to improve gastrointestinal motility in mouse feces (Fig. 5G–N). LGA significantly increased the level of isovaleric acid of constipated mice (Fig. 5L), while HGA significantly increased the level of hexanoic acid (Fig. 5M) and isohexanoic acid (Fig. 5N). In summary, the result suggests that the GABA may contribute to relieving constipation.

### 3.6 *L. rhamnosus* LRJ-1 enhanced GABA production of *B*. *uniformis*

**Fig. 6:**
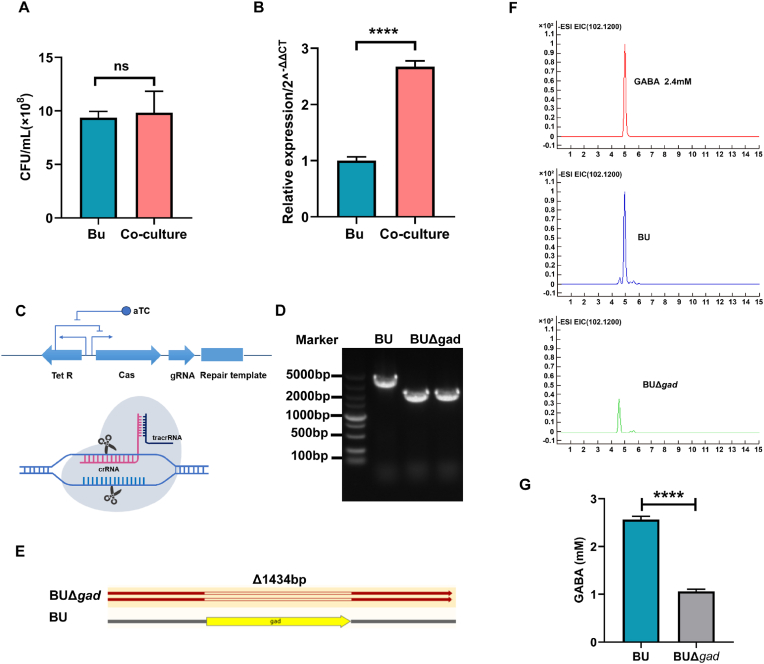
***L. rhamnosus* LRJ-1 enhanced GABA production from *B*. *uniformis* ATCC 8492.** (A) The growth profiles of *B*. *uniformis* ATCC 8492 co-cultured with *L. rhamnosus* LRJ-1. (B) The relative expression level of glutamate decarboxylase encoding gene *gad* co-cultured with *L. rhamnosus* LRJ-1. (C) Schematic diagram of CRISPR-FnCas12a system. (D) Colony PCR results from *gad* deletion mutants generated with CRISPR-FnCas12a system. (E) Sequencing results of the genotypes of the WT and Δ*gad* strains. (F) The content of GABA production in the medium of the WT and Δ*gad* strains determined by LC/MS. The standard curve of GABA is presented in the top. (G) Quantitative results of GABA content of the WT and Δ*gad* strains. ∗∗∗∗p < 0.0001 as determined by *t*-test through GraphPad Prism 8. ns: no significance.

As *B*. *uniformis* was the most enriched *Bacteroides* species (Fig. 3E) caused by *L. rhamnosus* LRJ-1 treatment, a *B*. *uniformis* ATCC 8492 (BU) strain was used to investigate its role in alleviating constipation. Firstly, we investigated the influence of *L. rhamnosus* LRJ-1 on the growth and GABA production of BU. *L. rhamnosus* LRJ-1 and BU were co-cultured *in vitro*, and the result shows that *L. rhamnosus* LRJ-1 did not promote the growth of BU (Fig. 6A). Previous study reported that Gad is the major microbial enzyme for GABA synthesis (Strandwitz et al., 2019), and GABA-producing pathways are actively expressed in *Bacteroides* (Strandwitz et al., 2019). Accordingly, we sought to explore whether Gad activity of BU was affected by *L. rhamnosus* LRJ-1. As indeed, *L. rhamnosus* LRJ-1 significantly increased the transcriptional activity of Gad in BU (Fig. 6B). To further investigate the effect of Gad in BU on GABA production, a GABA-synthesis-deficient strain of BU mutant (BUΔ*gad*) was generated using CRISPR-Cas genome-editing system (Fig. 6C–E). The GABA production of BUΔ*gad* was significantly decreased compared to BU (Fig. 6F, G). This result suggests that *L. rhamnosus* LRJ-1 can enhance the GABA production of *B*. *uniformis* by improving transcriptional activity of Gad.

### 3.7 GABA is responsible for *B*. *uniformis* mediated alleviation of constipation

**Fig. 7:**
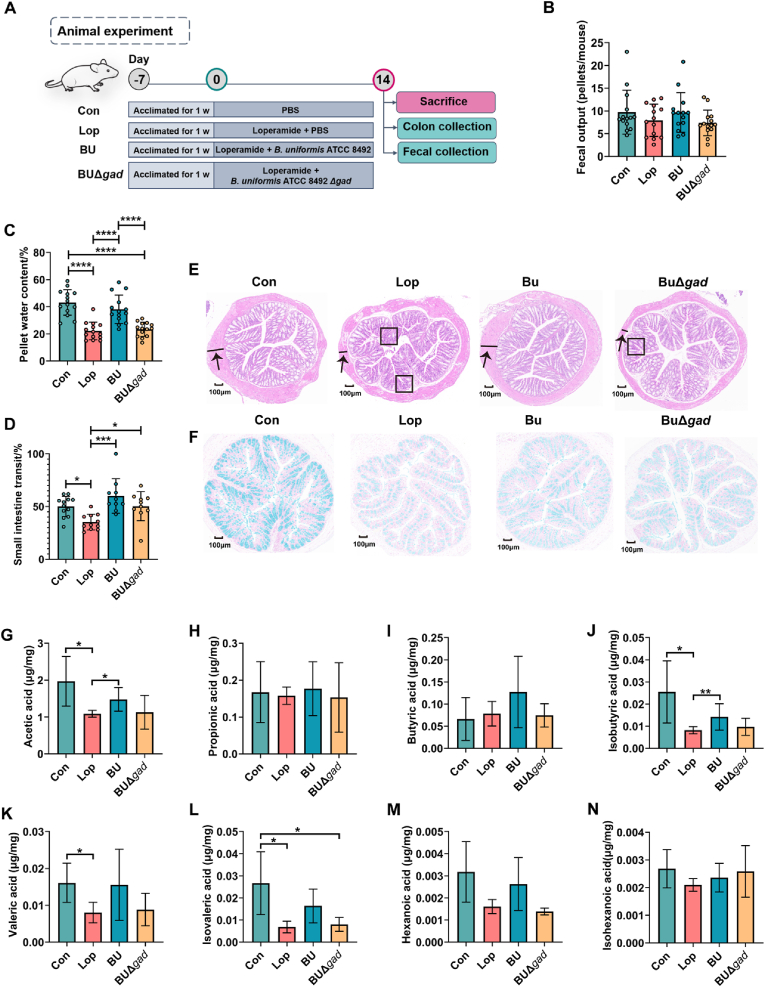
**The GABA is responsible for *B*. *uniformis* mediated alleviation of constipation.** (A) The experimental design. The picture of the mouse was created with BioRender.com. (B–D) The effect of *B*. *uniformis* ATCC8492 and *B*. *uniformis* ATCC8492 Δ*gad* on (B) fecal output, (C) pellet water content, (D) small intestine transit in loperamide induced constipated mice. (E) The effect of *B*. *uniformis* ATCC8492 and *B*. *uniformis* ATCC8492 Δ*gad* on histological morphology of colon by hematoxylin & eosin staining. (F) The effect of *B*. *uniformis* ATCC8492 and *B*. *uniformis* ATCC8492 Δ*gad* on colonic mucin by alcian blue staining. (G–N) The effect of *B*. *uniformis* ATCC8492 and *B*. *uniformis* ATCC8492 Δ*gad* on the contents of fecal short chain fatty acids by GC/MS, including (G) acetic acid, (H) propionic acid, (I) butyric acid, (J) isobutyric acid, (K) valeric acid, (L) isocaleric acid, (M) hexanoic acid and (N) isohexanoic acid. Con: control; LOP: loperamide; BU: *B*. *uniformis* ATCC8492. BUΔ*gad*: *B*. *uniformis* ATCC8492 Δ*gad*. ∗p < 0.05, ∗∗∗p < 0.001, ∗∗∗∗p < 0.0001 as determined by one-way ANOVA (B–D) or Wilcoxon signed-rank test (G–N). The black arrows indicate the muscle thickness. The black box indicates the inflammatory infiltration.

To confirm the beneficial effects of the enriched *Bacteroides*, BU and BUΔ*gad* was used to treat constipated mice to test the effect of BU and the GABA produced by BU for alleviating constipation as outlined in Fig. 7A. Compared with the Lop group, BU significantly improved the pellet water content and small intestine transit in constipated mice (Fig. 7C, D), while constipated mice orally treated with BUΔ*gad* exhibited little improvements in pellet water content (Fig. 7C). Moreover, decreases of pellet water content and small intestine transit were observed in BUΔ*gad* group compared to BU group, indicating a far less protective effect against constipation of BUΔ*gad* (Fig. 7C, D). Colonic tissue showed more intensive infiltration of inflammatory cell (as indicated by black boxes in Fig. 7E), thinner muscle layer (as indicated by black arrows in Fig. 7E), irregular colonic tissue structure and the decrease in production of goblet cells (Fig. 7F) in the Lop group compared to the Con group. BU intervention aggrandized the thickness of muscle layers and the number of goblet cells in colon (Fig. 7E, F), while BUΔ*gad* treatment did not improve the irregular colonic tissue structure of constipated mice, so did the thinner muscle layers and fewer goblet cells in colon tissues (Fig. 7E, F). Analysis of histomorphology further revealed the less protective effect of BUΔ*gad* compared to BU. The results of SCFAs contents showed that BU instead of BUΔ*gad* treatment obviously recovered acetic acid and isobutyric acid levels (Fig. 7G–N). These results indicated that GABA is responsible for BU-mediated alleviation of constipation.

## 4 Discussion

Our study highlights the interaction between exogenous probiotics and gut commensal bacteria to promote bacterially produced bioactive metabolites to modulate host function. Specially, we describe findings from an integrated multi-omics analysis of the gut metagenome, metabolome and host transcriptome in the context of constipation under *L*. *rhamnosus* LRJ-1 treatment. We further validated these findings by gene knockout and animal experiments. Collectively, we revealed that an increase in luminal *Bacteroides*-derived GABA level interacting with host GABAergic synapse, underlying *L*. *rhamnosus* LRJ-1 treatment, alleviated loperamide-induced constipation in mice.

The gut microbiota plays a central role in health and disease. An imbalance of gut microbiota can result in reduced bowel secretions, impaired colonic epithelial integrity and decreased gut motility, all of which are related to constipation (Zhang et al., 2021). Probiotics, such as *L*. *paracasei* (Cheng et al., 2023) and *L*. *rhamnosus* (Wang et al., 2020a) restored the balance of gut microbiota to alleviate the gut dysbiosis caused by constipation. Prebiotic chitosan oligosaccharides mitigated loperamide-induced constipation by modulating gut microbiota and increasing *Bacteroides* abundance (Zhang et al., 2021). Consistent with these studies, our results showed that a *L*. *rhamnosus* strain LRJ-1 alleviate constipation by enriching *Bacteroides* abundance, especially *B. uniformis*. Gut *Bacteroides* have been demonstrated benefit for host physiology. The *B. thetaiotaomicron* was found to regulate enteric neuronal innervation and neurogenic gastrointestinal activity (Aktar et al., 2020). The *B. uniformis* was reported to reduce hepatic steatosis in mice (Gauffin Cano et al., 2012). *B*. *vulgatus* and *B. dorei* were confirmed to attenuate atherosclerosis (Yoshida et al., 2018). Our finding expands previous reports that orally administration *B. uniformis* is beneficial for the alleviation of constipation in mice. Besides the above *Bacteroides* species, *Chlamydia abortus*, *Clostridiales bacterium*, *Klosneuvirus* KNV1, *Eubacterium siraeum*, *Chlamydia trachomatis*, *Parabacteroides goldsteinii* and *Bacteroidales* bacterium WCE2008 were also enriched significantly after *L. rhamnosus* LRJ-1 treatment. Among them, *P. goldsteinii* has been reported to alleviate obesity reducing serum pro-inflammatory cytokines and protecting the intestinal barrier (Wu et al., 2019). It meets the criteria of next generation probiotics (NGP) because of its protective effects on inflammation and obesity (O’Toole et al., 2017). However, we found that *Akkermansia*, a NGP with multiple health benefits in humans, was enriched in the constipated mice. It is consistent with the results reported by Wang et al. that the abundance of *Akkermansia* was significantly increased in the constipation model group (Wang et al., 2020a). Decreased *Bacteroides* but increased *Akkermansia* have also been observed in neurological diseases such as Parkinson's and Multiple Sclerosis (Mossad and Erny, 2020). *Akkermansia* also increases in some gastrointestinal diseases such as colorectal cancer, and it may only be beneficial in specific environments and at specific amounts (Hibberd et al., 2017). Moreover, *Turicibacter sanguinis*, *Paraclostridium benzoelyticum*, *Paeniclostridium sordellii*, *Eubacterium brachy, Streptococcus* sp HMSC034E12, *Clostridium perfringens* and *Clostridiodes difficile* were also significantly enriched in constipated mice. Among them, *C. perfringens* causes multiple diseases in humans due to its various toxins and virulence factors (Camargo et al., 2024). *C. difficile* can overgrow in the gut due to an imbalance of intestinal flora leading to *C. difficile* infection (CDI), which is a major cause of nosocomial infections (Tang et al., 2024).

The relative abundance of *Lactobacillus* in the control group (Con) of normal mice, the model group (Lop) of constipated mice, and the treatment group (LRJ-1) of constipated mice was 8.77%, 13.99% and 13.90%, respectively (Fig. 3D). Although there was no significant difference in the relative abundance of the *Lactobacillus* between the LRJ-1 group and the Lop group, the relative abundance of different *Lactobacillus* species varied significantly, particularly for *L*. *rhamnosus*. In the LRJ-1 group, *L. rhamnosus* accounted for 0.015% of the total *Lactobacillus* abundance, whereas in the Lop group, the proportions were only 0.0014% which are much lower than in LRJ-1 group (data not shown). We did not know why loperamide treatment resulted in the elevated abundance of *Lactobacillus* compared with control group, however, the proportion of *L. rhamnosus* in LRJ-1 group was much higher than in Lop group. We speculated that the administration of *L. rhamnosus* LRJ-1 exert a more pronounced effect on the *Bacteroides* genus compared to the *Lactobacillus* genus.

The gut motility is regulated by the interaction of gut microbiota, microbial-derived products, and host immune system and ENS (Mayer et al., 2014). The cross-talk between gut microbes and host is transmitted by various signal pathways involving a quantity of molecules. Hence, although metagenomic sequencing analysis revealed changes in the composition and functions of gut microbiota, the host-microbial metabolic pathways need to be analyzed to further understand the exact interaction between gut microbes and host. Recently, a multi-omics approach, integrated gut metagenome, metabolome and host transcriptome, was used to investigate microbial metabolites corresponding to host physiological mechanisms. Mars et al. found that purine metabolism is a novel host-microbial metabolic pathway in irritable bowel syndrome (Mars et al., 2020). Mayneris-Perxachs et al. reported that microbiota metabolites converging onto proline- glutamate- and GABA metabolism impacted depression (Mayneris-Perxachs et al., 2022). Accordingly, to clarify the gut-microbiota-targeted mechanisms of *L*. *rhamnosus* LRJ-1, we tested the alterations of intestine metabolites and host transcriptome. We found that *L*. *rhamnosus* LRJ-1 promoted the production of gut *Bacteroides*-derived GABA, which further elevated GABAergic synapse in host. It is reported that the enhanced GABAergic synapses results in increased MTL (Rajkumar 2021), and this is consistent with our results. Wu M et al. discovered that hesperidin ameliorates colonic motility in loperamide-induced constipated rats by elevating ADCY3, cAMP and PKA expression associated with GABAergic synapse (Wu et al., 2020). This is consistent with our results that *L. rhamnosus* LRJ-1 intervention increased AC and PKA genes expression in mice colon, which may promote colonic motility. Hence, the stimulation of GABAergic synapse caused by *L. rhamnosus* LRJ-1 treatment is important in ameliorating constipation.

GABA is the major neurotransmitter in body, and hence GABA-mediated neurotransmission regulates many physiological functions. Previous reports demonstrated that *L. rhamnosus* plays an important role in the bidirectional communication of the gut-brain axis to regulate depression and anxiety by GABA (Bravo et al., 2011). In addition to its role in affecting brain function, GABA also addresses a role in the modulation of gastrointestinal functions by affecting ENS. It plays a functional role in modulating gastrointestinal motility by regulating intestinal fluid and electrolyte transport (Hyland and Cryan, 2010). Previous work proposed that gut commensal *Bacteroides* can stimulate gut motility by increasing the synthetase gene expression of enteric GABA (Hooper et al., 2001). In our study, we identified that gut commensal *Bacteroides*-derived GABA is correlated to constipation relief. Moreover, both the gene knockout experiment and GABA administration experiment illustrated that GABA alleviated constipation in mice.

Park et al. reported that *Bifidobacteria* increase GABA production by genetically elevating Gad activity (Park et al., 2005). Sun et al. reported that co-fermentation of *Bifidobacterium adolescentis* and *L*. *paracasei* could produce more GABA than *L*. *paracasei* alone (Sun et al., 2023). In our study, we found that co-culture of *L. rhamnosus* LRJ-1 and BU increased *gad* expression in BU compared to that of BU alone, which was agreed with prior work. This finding further illustrates the interaction between *L. rhamnosus* LRJ-1 and gut commensal *Bacteroides* to promote GABA production for constipation alleviating. Importantly, commensal bacteria increasing intestinal GABA production allows for local delivery of GABA to the gastrointestinal tract and directly alter intestinal secretory activity.

SCFAs are able to stimulate gastrointestinal motility, intestinal transit and intestinal fluid secretion with consequent effect on constipation (Nicholson et al., 2012). In our study, BU significantly recover some of the fecal SCFAs in constipated mice. This result is also supported by the previous studies that chitosan oligosaccharides increased levels of fecal acetic-, propionic- and valeric acid in constipated mice (Zhang et al., 2021), and *L*. *plantarum* alleviated constipation in mice accompanied by increased levels of fecal acetic- and propionic acid (Li et al., 2015). It is reported that *Bacteroides* can hydrolyze polysaccharides to produce SCFAs (Trefflich et al., 2021; Singh et al., 2023). We proposed that the higher abundance of fecal SCFAs in our constipated mice was caused by the administration of BU. Our result showed that BU instead of *gad* deficient mutant and GABA treatment significantly recovered some of the fecal SCFAs in constipated mice, indicating that GABA may promote SCFAs production. The correlation between GABA and SCFAs production in gut has not been reported and needs further research.

In this work, the loperamide-induced constipation mice model was applied to prove that *L. rhamnosus* LRJ-1 has therapeutic potential for alleviating constipation. The Balb/c mice used in this study have a highly purified and stable genetic background, making them an ideal model for controlling variables in research. The mice were maintained under standard environmental conditions. All animals were uniformly fed the maintenance diet for mice to ensure their health and the consistency of experimental outcomes. Our results of the animal experiments were essentially free from the influence of the confounding factors. The loperamide-induced constipation mice model is commonly used to study the pathological mechanisms and potential treatments for constipation, and a two-week experimental period is often chosen to observe physiological changes (Zhang et al., 2023). This because loperamide primarily acts by activating μ-opioid receptors in the gut, inhibiting intestinal smooth muscle motility and water absorption, thus inducing constipation (Camilleri, 2011). Over time, μ-opioid receptors may become desensitized or downregulated, reducing the drug's efficacy. Although we have observed that a two-week treatment of *L. rhamnosus* LRJ-1 is efficient and safe, long-term effects and safety of *L. rhamnosus* LRJ-1 administration still need to be evaluated to understand its chronic impact on constipation and overall gut health.

Our study demonstrated that *L*. *rhamnosus* LRJ-1 promoted an increase in gut *Bacteroides*-derived GABA production, and alleviated constipation through GABAergic synapses in mice. Our results showed that taking either *L*. *rhamnosus* LRJ-1 or *B. uniforms* effectively alleviated constipation symptoms, indicating that both strains hold potential for treating constipation. As *Bacteroides* are recognized as the main GABA-producing bacteria in the gut (Strandwitz et al., 2019), we supposed that *L*. *rhamnosus* LRJ-1 and GABA producing *Bacteroides* could be further applied to human trials for constipation treatments. Furthermore, our results showed that direct administration of GABA also proved effective for constipation treatment. Oral GABA has previously been reported as feasible in a randomized trial examining its effects in children with diabetes (Martin et al., 2022), indicating that oral GABA could also be a potential therapeutic strategy for human constipation. Further research is required to determine the appropriate dosage and efficacy of GABA for constipation treatment.

## 5 Conclusions

In summary, we demonstrated the therapeutic effects and mode of action of *L. rhamnosus* LRJ-1 on constipation. *L. rhamnosus* LRJ-1 effectively increased fecal output number and fecal water content, and decreased colonic injury. *L. rhamnosus* LRJ-1 alleviated constipation in mice accompanied by the increased abundances of GABA in gut. A modified gut microbiota, characterized by a notable increase in the abundance of GABA-producing *Bacteroides* spp., was identified as a key factor for the beneficial effects of *L. rhamnosus* LRJ-1. We also found that *L. rhamnosus* LRJ-1 activated the host GABAergic synapses. Our findings reveal a specific exogenous *L. rhamnosus* LRJ-1-gut *Bacteroides*-host GABAergic synapse pathway for the alleviation of constipation. Furthermore, this work offers insights into the therapeutic mechanism targeting gut microbiota and their metabolite GABA to alleviate constipation.

## CRediT authorship contribution statement

**Tianqi Xia:** Conceptualization, Data curation, Writing – original draft. **Fuqing Huang:** Investigation, Methodology. **Fangfei Yun:** Validation, performing experiments. **Yayong Liu:** Validation, performing experiments. **Tianwei Wang:** Validation, performing experiments. **Siyue Wang:** Validation, performing experiments. **Sijie Jin:** Validation, performing experiments. **Xingwang Ma:** Validation, performing experiments. **Wenhan Wang:** Validation, performing experiments. **Jianzhuo He:** Validation, performing experiments. **Kunling Teng:** Project administration, Funding acquisition, Writing – review & editing. **Jin Zhong:** Project administration, Funding acquisition, Writing – review & editing, All authors contributed to the article and approved the submitted version.

## Ethics approval and consent to participate

The protocol for the animal experiment was approved by the animal ethical committee of the Institute of Microbiology, Chinese Academy of Sciences (approval number APIMCAS2022025), and Institute of Biophysics, Chinese Academy of Sciences (approval number SYXK2024222). All ethical requirements of the study were met. Informed consent was obtained from all participants at the beginning of the study.

## Data availability statement

The data of RNA sequencing in the current study is provided by National Microbiology Data Center. https://nmdc.cn/resource/en/genomics/sample/detail/NMDC20170562

The data for the gut metagenome is provided by National Microbiology Data Center. https://nmdc.cn/resource/en/genomics/sample/detail/NMDC20170553

## Funding

This work was supported by National Key R & D Program of China, China (2022YFA1304203, 2021YFC2103901); 10.13039/501100001809National Natural Science Foundation of China, China (U20A2066); Strategic Priority Research Program of the Chinese Academy of Sciences, China (XDA26040201).

## Declaration of competing interest

The authors declare that they have no known competing financial interests or personal relationships that could have appeared to influence the work reported in this paper.
